# Dr. Mackness on the Moral Aspects of Medical Life

**Published:** 1847-04

**Authors:** 


### Art. XI.

*The Moral Aspects of Medical Life, consisting of the 'Akesios' of Professor K. F. H. Marx. Translated from the German, with Biographical Notices and Illustrative Remarks.* By James Mackness, m.d., Member of the Royal College of Physicians, &c.—*London,* 1846. Sm. 8vo, pp. 348.

In the 19th Volume of this Journal we noticed Professor Marx's 'Akesios; a Glance into the Ethical Relations of Medicine.' Dr. Mackness has taken up this thin volume, and by adding a biography of each person to whom Professor Marx addressed his letters, and a running commentary, has swelled it into a goodly octavo. His first intention was to publish a translation only, but as he proceeded to scan the work, line by line, he was "struck with its remarkable condensation of style, and with the germs of noble thought which often lay buried in a single sentence." So Dr. Mackness determined to make these germs germinate, and to view on all sides certain interesting subjects of which Professor Marx only takes a glance; to compare, contrast, illustrate them.

The biographical notices are all selected; some of them are translated from the collection of 'Eloges Historiques,' and others from articles in the 'Dictionnaire Universelle.'

The biographical notice of Stieglitz is derived from a recent memoir of that physician by the author of 'Akesios,' entitled 'Zum Andenken an Johann Stieglitz.' The details of the professional life and character of the twelve distinguished physicians and surgeons to whom Marx addresses his epistles are generally interesting and instructive.

The 'Remarks' which follow each letter are, on the whole, excellent, and creditable alike to the head and the heart of the commentator. Some little blemishes a critical eye can easily detect in them. They are somewhat too much like sermons; too much in the firstly, secondly, thirdly vein to read well, at least when regarded in a mere literary point of view. The great member of quotations is a further drawback. "'Besides the qualifications,' says Gregory." "'Woe to the physician,' says Simon, 'who,'" &c. "We much admire Percival's view of this subject; he says," &c. "Here we may cite a passage from Dr. Simon. 'If the masters of the science,' says he," &c. Now, all this "he says and she says" gives the book a gossiping air; and the numerous phrases of a peculiar stamp so liberally interspersed through the remarks, make us involuntarily think of an evangelical tea-table. We do not use the phrase in an offensive sense, and merely wish thereby to designate that class of persons who, thoroughly devoted to religion, get their conversation tinctured with biblical phrases, and contract a sermonizing style. We honestly think it to be matter of regret that Dr. Mackness's work should be thus tinctured, for it abounds with just sentiments and pure morals, and the effect of the peculiarity mentioned is to render these valuable qualities less attractive. Many of the quotations are eminently beautiful, and several of the views expressed by the commentator are good and well given: they would have been indeed much better if Dr. Mackness had boldly pushed off and quoted less. For example, Dr. Mackness would have written much better on cheerfulness as a remedy, if he had trusted himself more, and the "late excellent Rev. Robert Anderson of Brighton" less.

We shall now give a few extracts as examples of Dr. Mackness's style, and on some of them we may offer occasional remarks.

"There is one trait in the character of Hallé which, however much we may admire the spirit which prompted it, we cannot approve, and that is his reluctance to receive fees, even from those who could well afford to pay. It is true that his ample fortune might be quite sufficient to supply all the wants of his family, and, therefore, such a line of conduct was attended with no great sacrifice; while to his benevolent mind it doubtless afforded him the purest enjoyment. But did none others suffer from such a line of policy? Was the medical profession so affluent in his day in Paris, that there were no men of energy and talent belonging to it, who, without property, without friends, felt their spirits broken, their hearts sickened, for want of professional success? Such is too often the case in England, and doubtless the same in France." (p. 66.)

This is all very correct; so also is the adoption of Percival's views that the poorer clergy should be attended gratuitously, as well as all the members of the medical profession and their immediate families. But we should have been glad to have seen Dr. Mackness raise the question as to the fees to be demanded from those persons who are "decent people" but not poor; persons with large families and limited incomes, but "respectable." Is the same fee to be taken or demanded from these as from the wealthy? and if not, on what principles, or according to what proportion is the abatement to be made? Is the physician to refuse his advice to the decent person? Or how is it to be given? We believe that a just determination on this point can only be come to, on weighing the merits of individual cases. But we trust the medical profession will never forget their glorious mission as helpers of humanity, and think of their duty first, their interest afterwards. In the class of persons here referred to, we often find the most liberal disposition in regard to fees, and at the same time a becoming pride in coming under obligations. A wise and good physician will generally find little difficulty in deciding as to the proper conduct. A fee may be once, or occasionally, taken to destroy the unpleasant sense of obligation; and excuses may easily be found for paying friendly visits, more or less frequently, according to the nature of the disease and other circumstances. In some cases of the kind under consideration, unquestionably, fees should be entirely declined; and the heart that is really kind, will readily prompt excuses that will not give offence. In all cases, and under all circumstances, let the physician remember, that liberality carried even to excess is but a venial fault, while the reverse is a professional sin of the deepest dye, degrading to the individual, and dishonoring to the class to which he belongs.

The letter to Albert Thaer affords an opportunity for the discussion of medical scepticism. With reference to the present direction of the current of change in the profession, Dr. Mackness observes:

"An important change appears in the present day to be taking place in the minds of many of the medical body, especially in its higher sections—a change favorable to greater reliance on the powers of Nature and on simple hygienic measures. Whilst we foresee from these changes a better era in the history of medicine, there is also some reason to fear the increase of medical scepticism, for which, in Simon's view, homœopathy itself is only a decent name. Dr. Robert Williams is mentioned, in an oration to the Medico-Chirurgical Society, as having had no faith in medicine. One can sympathize with men who, like Thaer, Haller, and others, declined practice from an over-scrupulousness of mind and dissatisfaction with the state of the science, but it seems hardly consistent with moral honesty to continue to practise with such views of the medical art." (p. 138.)

We fully agree with the opinion here expressed; but we suspect there are very few persons indeed who have no faith in medicine. Even the apparently greatest sceptics are often the most credulous. Thus a strong-minded man will sneeringly refuse the advice and assistance of a hospital surgeon for a wound or an ulcer, and trustingly consult an old soldier returned from the wars. And so also with such men as Thaer and Haller; it is not that they doubt the value of medical art and science, but rather the value of that experience which is so dogmatically thrust before the public by evidently incompetent persons. They have, in short, no certainty of many of the facts, and still more of the theories, and hence they despair of success, and practise without hope. This indicates a want of sound common sense and decision of character. The physician may be allowed to doubt, that he may the better arrive at truth, for, in fact, there can be no true faith where doubts have never entered.

In his remarks on the letter to Boerhaave, Dr. Mackness reverts to doctrines recently promulgated and advocated in this Journal, honoring us, (we are bound to say) by quoting us more than once.

"From nomenclature the letter proceeds to touch on practice, and our author acknowledges that, whilst there has been a departure from simplicity in the former, there has been an approximation to it in the latter; and he instances the greater simplicity in modern practice of prescriptions, of surgical treatment, and of obstetric practice, and proceeds to notice the greater prominence now given to moral and hygienic measures in medicine. Amongst the thinking part of the profession there is, it is well known, a feeling in favour of the adoption of a rational system of hygiene and regimen in preference to active therapeutics. It is becoming better understood that many diseases run a natural course and terminate in a particular manner; and that the most that art can do is to watch their progress, and place the system in that position in which the malady is likely to terminate favorably." (p. 337.)

We believe there is no topic of medical ethics untouched in this volume; at least no important topic. The various questions of morals and manners which arise out of the biographical sketches, and out of the sentiments expressed in the letters, enable Dr. Mackness to be discursive, but finally comprehensive. We cannot undertake to indicate, even in a bare outline, the principal questions; our readers will, we are sure, be both interested and instructed by the volume, and we, therefore, recommend it to them, and strongly. Dr. Mackness has done a decided service to the profession in compiling this work. We trust he is only the pioneer to open the way to a still greater undertaking of the same kind.

While thus approving of his work generally, and with the most friendly feelings towards the author personally, we cannot quite forego that less agreeable task which forms part of our editorial duty, of noticing the literary demerits of the volume. We regret to say that it contains a good many inelegancies of style, and a considerable number of imperfections of translation. We observe errors even in those few portions of which we gave a translation in our review of the original work, and which we have reason to know gave satisfaction to the author with a solitary exception.*

As Dr. Mackness is a reader of this Journal, our notice could hardly have escaped him, and seeing that our version differed from his, he ought, we think, to have assured himself fully that his was correct. Some of Dr. Mackness's errors really misrepresent his author. Thus in the opening sentence of the letter to Stieglitz, Professor Marx is represented as saying: "Since the 31st of October 1840, when you bade farewell to these earthly scenes, not merely my writing-paper, but every day of my life has worn a mourning edge." But Professor Marx had no such connexion with Stieglitz as would warrant the use of black-edged paper, and, in fact, he did *not* use it, and says so. "Since the 31st, of October, 1840, when you said farewell to the earthly, my days, and not my writing-paper, have had a margin of mourning." We willingly allow that Professor Marx is not an easy writer to translate, but surely where the work was already done to Dr. Mackness's hand he might have appropriated it. Some of the expressions lose all their pith in the translation, and are even made ludicrous. For example, Dr. Mackness translates "Die Gedankenscheu ist so unheilbar wie die Wasserscheu," into "Bashfulness is as incurable as hydrophobia," and on this he hangs a comment, a quotation from Cowper, and an extract from this Journal. Yet Professor Marx never thought that bashfulness is so incurable. The aphorism should be rendered "the dread of thinking is as incurable as (hydrophobia) the dread of drinking." Another mistranslation is "physicians are born honorary members of all *human* societies," whereas it should have been, and as Dr. Mackness might have learnt from our translation, *humane* societies, the point having reference to societies instituted for the preservation of persons

* For "he who has no theory cannot be practical," we ought to have said, "—— is not *therefore* practical.

from death, as our own Royal *Humane* Society. Another example of incorrectness is one which concerns our own *métier.* Dr. Mackness translates an aphorism thus: "Reviewers are like the scourge, as the tails of comets, they frighten the weak, but do the strong no harm." Now the point in this aphorism is in likening reviews (not reviewers) to birch-rods, to which handy instruments of correction the tails of comets are not, in form, at least dissimilar, and not unlike in their operation on weak minds, in being considered scourges. The passage should have been, "reviews are just such rods as the tails of comets; they frighten the silly, but don't alarm the strong." Let some of our thin-skinned friends ponder this aphorism, and wince less when their faults are pointed out.

In addition to these actual errors, (which we have taken at random) we observe that several passages are so done into English as to read obscurely, or at least inelegantly. We subjoin one more example:

> "For the dying there is an euthanasia, for the mourner a visit of condolence; but who concerns himself about the suffering physician? And yet he has most frequently to experience, that in bereavements the tears of survivors become like aquafortis to the soul, and that powerlessness to save others curdles, as it were, his own blood."*

There are two points here, the one is the play on *scheiden,* which signifies separation, and figuratively death, and *scheide*wasser, which is aquafortis used in etching;—the English verb to etch, being derived from the German aetzen, to corrode by acid or caustic. The other is in the word ohnmacht, or fainting; figuratively the heart bleeds from grief; fainting (the weakened heart) checks a hemorrhage, so the consciousness of *powerlessness,* the *literal* meaning of ohnmacht, may check the feelings of sorrow. The version we would suggest might run thus:

> "There is heaven for the dying, and visits of condolence for the mourning survivors; but who troubles himself about the sorrowing physician? And yet the latter has the most frequently to experience and to feel that corroding death, with tears for aquafortis, etches sorrows on the soul; and that 'tis only a conviction of his powerlessness to save, that relieves his bleeding heart, as the powerlessness of a swoon checks the bleeding from a wound."

With such a form of expression, every practitioner will call to mind how deeply, when he has stood by the bed of a dying patient, he has felt his inability to save or even to relieve, and that his only consolation was a thorough confession of his utter helplessness.

This volume is affectionately dedicated by the author to his friend Dr. Archibald Robertson, of Northampton, in terms of the justest eulogy, as all who know him will avouch; a physician, "who, in his intercourse with his brethren and the public, has, during a long professional career, honorably exemplified the principles maintained in the following pages."

* "Für die Sterbenden hat man eine Euthanasia, für den Trauernden Condolenzbesuche; wer kümmert sich um den leidtragenden Artz? Und doch hat dieser am häufigsten zu erfahren und zu empfinden, dass beim Scheiden die Thränen zu Scheidewasser für die Seele werden, und dass die Ohnmacht das Bluten stillt." (Akesios, p. 21.)

